# A DFT Study of CO_2_ Hydrogenation on Faujasite‐Supported Ir_4_ Clusters: on the Role of Water for Selectivity Control

**DOI:** 10.1002/cctc.201600644

**Published:** 2016-06-23

**Authors:** Bartłomiej M. Szyja, Daniel Smykowski, Jerzy Szczygieł, Emiel J. M. Hensen, Evgeny A. Pidko

**Affiliations:** ^1^ Department of Chemical Engineering and ChemistryEindhoven University of TechnologyDen Dolech 25612MBEindhovenThe Netherlands; ^2^ Division of Fuels Chemistry and TechnologyFaculty of Chemistry, Wrocław University of Technologyul. Gdańska 7/950-344WrocławPoland; ^3^ Institute of Complex Molecular SystemsEindhoven University of TechnologyDen Dolech 25612MBEindhovenThe Netherlands; ^4^ Faculty of Mechanical and Power EngineeringWrocław University of TechnologyWybrzeże Wyspiańskiego 2750-370WrocławPoland

**Keywords:** carbon storage, density functional calculations, hydrogenation, reaction mechanisms, zeolites

## Abstract

Reaction mechanisms for the catalytic hydrogenation of CO_2_ by faujasite‐supported Ir_4_ clusters were studied by periodic DFT calculations. The reaction can proceed through two alternative paths. The thermodynamically favoured path results in the reduction of CO_2_ to CO, whereas the other, kinetically preferred channel involves CO_2_ hydrogenation to formic acid under water‐free conditions. Both paths are promoted by catalytic amounts of water confined inside the zeolite micropores with a stronger promotion effect for the reduction path. Co‐adsorbed water facilitates the cooperation between the zeolite Brønsted acid sites and Ir_4_ cluster by opening low‐energy reaction channels for CO_2_ conversion.

## Introduction

Development of new technologies that utilise carbon dioxide as a C_1_ building block for the production of value‐added chemical and fuels is considered as one of the key steps towards the green and sustainable chemical industry. Reductive transformation of carbon dioxide attracts particular attention of the scientific community as it provides a method to store renewable energy in chemical bonds.^1^ For example, catalytic hydrogenation of CO_2_ is one of the pivotal elements of the so‐called methanol economy advocated by the Nobel laureate George Olah.^2^ Methanol is a key chemical that can potentially substitute fossil fuels and, at the same time, serve as a versatile platform for the production of a wide range of chemicals.^2^, ^3^

Catalytic hydrogenation of CO_2_ can proceed along three paths, namely (i) hydrogenation to formic acid (FA) or methanol, (ii) methanation to CH_4_ and (iii) reduction to CO. The last two processes are usually performed in the gas phase in the presence of heterogeneous catalysts, whereas the direct hydrogenation reaction for the production of FA from CO_2_ is performed in the liquid phase.^3^

The possibility to convert CO_2_ to methane (methanation process) in the presence of bulk metals was discovered by Sabatier at the beginning of the 20th century. This discovery was followed by extensive research focused on the optimisation of both catalyst and process.^4^ The key step in this reaction was found to be the dissociation of the CO_2_ molecule on a metal surface.^5^ A representative example of a supported metal catalyst for such a process is zeolite Y‐supported Ni/Ce nanoparticles.^6^ The high reactivity of this catalysts was attributed to the combined effect of ceria to promote the initial CO_2_ dissociation and the subsequent fast hydrogenation catalysed by Ni.^6^

The catalysts formally having lower hydrogenation activity form the basis for the reductive conversion of CO_2_ to CO, which can then be utilised as a conventional C_1_ building block in well‐established processes such as methanol synthesis, Fischer–Tropsch synthesis, or carbonylation.^7^ With respect to the latter, Losch et al. have recently reported an easy and green carbonylation technology involving CO generated in situ upon the decomposition of FA over Brønsted acidic H–ZSM‐5 zeolite at mild conditions.^8^

Another route involves the direct hydrogenation of CO_2_ with H_2_ without prior CO_2_ dissociation. This reaction is central to a technology for H_2_ storage in the form of a liquid fuel.^9^ Significant progress in the direct CO_2_ hydrogenation has been made in recent years with the development of new highly efficient and robust homogeneous transition‐metal catalysts.^3^ In particular, Ru^10^, ^11^ and Ir‐based^12^, ^13^ homogeneous catalysts attract much attention in view of their exceptional activity and stability in the catalytic CO_2_ hydrogenation. Very recently, it was shown that the Ir‐based pincer catalysts do not only have an exceptional catalytic performance in CO_2_ hydrogenation to formates,^12^ but also are able to promote the reductive cleavage to CO,^14^ which involves a cooperative action between the transition metal centre and a neighbouring Brønsted acid functionality of the ligand.11a

FA is not only a promising energy carrier, but also an important intermediate for the chemical industry. The introduction of more sustainable and efficient technologies for its production from renewable sources such as CO_2_ is desired. Although homogeneous catalysts show great potential for practical reversible H_2_ storage technologies,^9^, ^15^ commercial FA synthesis in this way is hampered by challenges in catalyst regeneration and separation of reactants.^7^ Therefore, an efficient and selective heterogeneous catalyst for CO_2_ hydrogenation to FA is desired.

The first example of FA synthesis from CO_2_ and H_2_ was reported in 1914 by Bredig and Carter, who demonstrated the possibility of catalytic hydrogenation of CO_2_ and aqueous alkali carbonates to FA under mild conditions in the presence of bulk Pd.^16^ After approximately 100 years from these initial observations, a catalyst formulation of the 21st century that also utilised Pd as the key component has been reported. Bi et al. have recently described a highly active system for reversible CO_2_ hydrogenation based on a reduced graphene oxide supported Pd catalyst.^17^ Metal oxide supported Au nanoparticles have also been studied for the liquid‐phase CO_2_ hydrogenation.18a,18b In addition, grafting of Ir and Ru complexes onto mesoporous supports has been considered as a promising strategy towards heterogeneous CO_2_ hydrogenation catalysts.18c,18d,18e

Zeolites represent an important class of aluminosilicate materials suitable for the design of new efficient heterogeneous CO_2_ hydrogenation catalysts. They combine high stability, a well defined porous system and relatively low cost with an outstanding degree of chemical tunability. The isomorphous substitution of a part of the silicon atoms in their framework for aluminium generates a local negative charge on the lattice that is compensated by an extra‐framework cation. The compensation of such negative charges by protons results in the formation of strong Brønsted acid sites. Alternatively, these anionic centres together with the surrounding basic lattice oxygen centres can form a suitable ligand environment for the stabilisation of a wide variety of cationic species with desirable catalytic properties.^19^ Furthermore, zeolites allow the construction of reactive environments featuring multiple reactive centres of different chemical nature inside their molecular‐sized cages and channels. These reactive sites can act cooperatively to enable low‐energy reaction paths.^20^ Such an active site cooperativity is analogous to the related reactivity concept in homogeneous catalysis.11a

Zeolite‐based materials have already been considered as potential catalysts for CO_2_ transformations.^21^ The hydrogenation of CO_2_ over zeolite Brønsted acid sites (BAS) and extra‐framework alkali cations has been computationally investigated by Chan and Radom21a,21b who proposed a possibility for the efficient conversion of CO_2_ by acidic zeolites through a concerted mechanism. The reaction involved protonation of the adsorbed CO_2_ by zeolite BAS and simultaneous heterolytic dissociation of H_2_ to produce formate and regenerate the BAS. The CO_2_ activation and the formation of the formate species is a key step in the overall process. It can be facilitated by more reactive hydride anions formed upon the H_2_ dissociation over a transition‐metal site. A transition‐metal species stabilised by the zeolite lattice, which at the same time provides mobile protons necessary to close the catalytic cycle of the FA formation,^10^ is a promising candidate as a heterogeneous system for CO_2_ hydrogenation.

The stabilisation of transition metal clusters inside the zeolite pores has been extensively studied theoretically by the groups of Rösch and Vayssilov^22^ and experimentally by Gates and co‐workers.^23^ In particular, molecular‐sized Ir_4_ clusters stabilized in faujasite (FAU) zeolites were found to be highly active in hydrogenation catalysis.23c–23e The possibility of the hydrogen spillover between the Ir_4_ clusters and the zeolite framework has been proposed as an important feature of the Ir_4_/FAU systems relevant to their catalytic performance.22d Such molecular‐sized clusters show distinctively different chemical properties and catalytic reactivity from the bulk nanoparticulate catalytic ensembles commonly employed in conventional heterogeneous catalysis.^24^ Because of their small size, the clusters do not possess a developed band structure such as that observed in transition‐metal nanoparticles and rather resemble the sites encountered in homogeneous catalysts.

The unique ability of Ir_4_/FAU to create a reactive environment inside zeolite micropores combining relatively strong BAS^20^ and multiple hydride species on the transition metal cluster^22^, ^23^ renders this system a promising catalyst for reductive transformations of CO_2_. In this work, we carry out a periodic density functional theory study on a realistic FAU zeolite model to evaluate the potential of this system for CO_2_ hydrogenation. We investigate the possibility to control the reaction path of CO_2_ conversion and analyse the effect of co‐adsorbed water molecules inevitably present inside the zeolite micropores on the catalyst performance and the reaction mechanism.

## Computational Details

### Models

**Figure 1:**
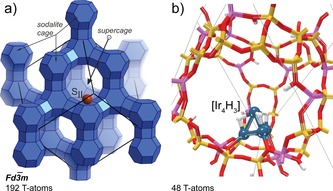
a) The topology and location of SII extra‐framework site in faujasite zeolite. Thin lines represent the crystallographic Fd−3m faujasite unit cell. The brown sphere represents the location of active cluster. b) The supercage of the small‐size triclinic faujasite unit cell employed in this study. Oxygen atoms are marked in red; Si and Al substitutions in the frameworks are marked in yellow and pink respectively. The Ir_4_H_3_ cluster is represented with blue (Ir) and white (H) spheres.

The reaction paths were analysed in the framework of density functional theory (DFT) using a periodic faujasite (FAU) zeolite model with a Si to Al ratio of 2.42 representing the lattice composition of zeolite Y. Similar to our previous studies,^25^ the low‐symmetry triclinic FAU unit cell was used as a model. The optimised cell parameters were *a*=*b*=*c*=17.51 Å and *α*=*β*=*γ=*60°. The negative lattice charges owing to substitution of the framework Si^+4^ by Al^+3^ ions were compensated by the extra‐framework iridium cluster with an overall charge of +2 and protons. The Ir_4_ cluster compensated for the negative charge of two lattice Al centres, whereas the remaining 12 anionic lattice sites present in the framework were compensated by H^+^ giving rise to Brønsted acid sites. The faujasite structure and the respective model with embedded Ir_4_ cluster are shown in Figure 1. The initial structure and location of the Ir_4_ unit was constructed on the basis of extensive experimental^22^ and computational data^23^ previously reported for this system. The current zeolite model represents a realistic system, with full periodic structure of the FAU framework that accounts for all the effects present in the confined space of the zeolite channels. The only major approximation used in this work is the assumption of the defect‐free infinite periodic structure of the zeolite.

The reactive site was represented by a partially hydrogenated Ir_4_ cluster (Figure 1 b). Previous experimental^23^ and theoretical22a,22b,22c,22d studies showed that such species preferentially adopts a tetrahedral configuration inside the zeolite. The formation of alternative planar structures for such species has never been reported before. Our computational results are in line with the previous findings. Chemical transformations catalysed by the zeolite‐stabilised Ir_4_ cluster result in geometrical perturbations that are limited to minor distortions caused by the interactions with the reactants and do not lead to any significant change in the overall Ir_4_ shape. The initial cluster bears three hydride ligands and is stabilised at the SII six‐membered‐ring cation site, where it compensates for the charge of two anionic [AlO_4_]^−^ units in the FAU supercage. Such a configuration allowed us to assess different mechanisms of CO_2_ transformations initiated by the chemisorption of CO_2_ to the under‐coordinated Ir centre at the vertex of the cluster. This activation route was identified as the preferred one among a series of alternative mechanisms considered by the initial computational screening. Besides the mechanistic paths discussed in detail below, the initial computational assessment included mechanisms such as the outer sphere CO_2_ hydrogenation and its activation at the edge sites of Ir_4_. The structural models for the respective intermediates either converged to the species involved in the main paths discussed below or showed prohibitive thermodynamics of elementary steps and therefore were not considered further.

The results obtained in the course of this study indicate that even the smallest amount of water can significantly affect the reactivity and selectivity of the investigated system. In view of the complexity of the reaction mechanisms considered and the size of the systems, the current mechanistic analysis was limited to the static relaxation simulations. The investigation of the effect of higher water content, although of great interest, was beyond the scope of the current study and would require a prolonged sampling of the Free Energies by using Molecular Dynamics simulations, which would be prohibitive in terms of computational demand for the analysis of all relevant paths.

### Methods

The DFT calculations were performed using the Vienna Ab Initio Simulation Package (VASP).^26^ The generalised gradient approximated PBE exchange–correlation functional was used.^27^ The electron–ion interactions were described by the projected augmented waves (PAW) method.^28^ The Brillouin zone sampling was restricted to the *Γ* point.^29^ The energy cut‐off was set to 500 eV. The convergence was assumed to be reached if the forces on each atom were below 0.05 eV Å^−1^. Such computational parameters provide a good compromise between the accuracy and computational demands for studying chemical processes in zeolite micropores as we have demonstrated in our previous successful studies on related subjects.^30^ A modest Gaussian smearing was applied to band occupations around the Fermi level, and the total energies were extrapolated to σ→0. The nudged‐elastic band method (NEB)^31^ was used to determine the minimum energy path and to locate the transition state structures along the CO_2_ hydrogenation paths. The nature of stationary points was confirmed by determining the vibrational frequencies using the finite difference method. Small displacements of 0.015 Å were used to determine the numerical Hessian matrix. Transition states were identified by verifying the occurrence of an imaginary frequency along the expected reaction coordinate.

## Results and Discussion

**Figure 2:**
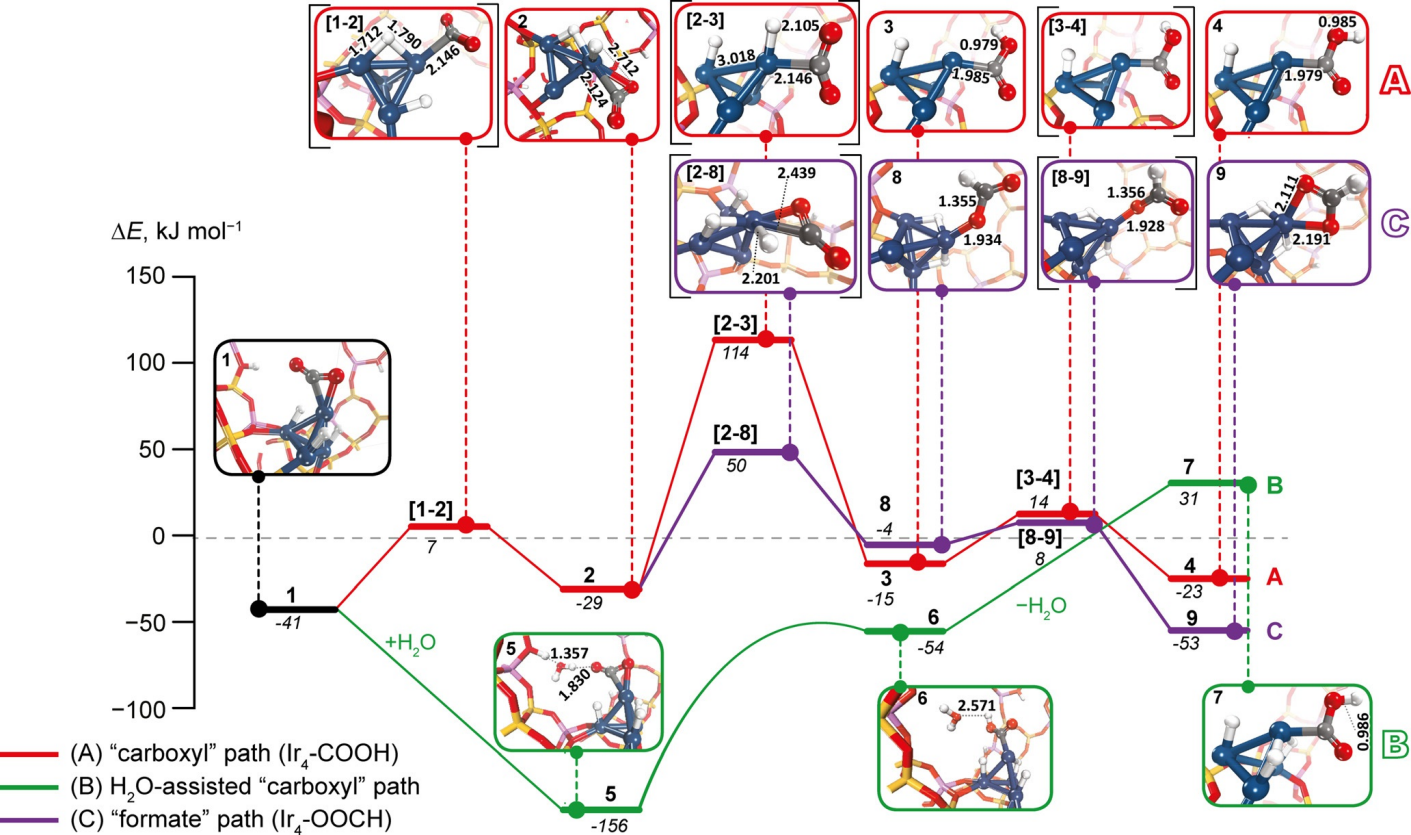
Reaction energy diagram for the first H transfer to the CO_2_ molecule. The relative energies for each state (in italic) are given in kJ mol^−1^ with respect to the sum of the energies of free reactants. The water‐free (**A**) and water‐assisted (**B**) path to carboxyl are shown in red and green, respectively. Pathway (**C**) via a formate intermediate is shown in purple. Transition‐state structures are denoted by the square brackets. The Ir cluster is represented by blue spheres and H is shown as white spheres. C, O, Si and Al are shown in grey, red, yellow and pink, respectively.

The reductive transformations of carbon dioxide by Ir_4_/FAU are initiated by its chemisorption to the under‐coordinated Ir centre at the vertex of the Ir_4_ cluster. This step proceeds with a negligible barrier and is exothermic by 41 kJ mol^−1^. The adsorption of CO_2_ is accompanied by the change of the hybridisation from the linear sp to the sp^*2*^‐type resulting in the formation of a strong *η* ^2^‐(C,O)‐type adsorption complex **1** with the under‐coordinated Ir centre (Figure 2). The distance from the non‐coordinated oxygen of the adsorbed CO_2_ to the nearest Brønsted acid site of the zeolite support is rather large (4.36 Å).

Further hydrogenation of the adsorbed CO_2_ molecule can take place by Ir‐bound hydrides. The optimised structures of key reaction intermediates and the relevant reaction energy diagram are summarised in Figure 2. Prior to CO_2_ hydrogenation, a reactive hydride ligand migrates from the base of the cluster to the Ir atom bound to CO_2_. This step **1**→**2** is almost thermoneutral (Δ*E*=12 kJ mol^−1^) and shows a low activation barrier (*E* ^≠^) of only 48 kJ mol^−1^. The subsequent attack on the adsorbed CO_2_ by Ir−H to form Ir−COOH (**3**) is much more difficult (Δ*E*=14 kJ mol^−1^, *E* ^≠^=143 kJ mol^−1^). The highly activated nature of this reaction originates from the need of repolarisation of the reactive species—the protonation of adsorbed CO_2_ is done by a formally anionic hydride. Subsequent facile (Δ*E*=−8 kJ mol^−1^, *E* ^≠^=29 kJ mol^−1^) rotation of the hydroxyl group leads to **4**, which is the Ir_4_−COOH isomer that is ready for further transformations along the hydrogenation reduction paths.

Alternatively, the −COOH intermediate may be formed by proton transfer from the zeolite lattice. This path is however very unfavourable (*E*_act_=421 kJ mol^−1^), because of the large distance for the proton transfer. We explored the possibility of facilitation of the H^+^ transfer step by a water molecule. The corresponding energy diagram for the water‐assisted reaction path **B** is shown in green in Figure 2. To model this mechanism we placed a water molecule between the acidic centre of the faujasite and the Ir_4_ site *(E*_ads_=115 kJ mol^−1^, **1**+H_2_O→**5**, Figure 2). One proton of the water molecule in **5** is directed towards the oxygen atom of the CO_2_. The subsequent transfer of the proton to form an Ir‐bound−COOH species **6** is an endothermic (102 kJ mol^−1^) barrierless reaction.

Water desorption at the next step destabilises the system by 85 kJ mol^−1^. This step may or may not be needed depending on which further reaction steps are assumed. The desorption is described here for completeness as it does not signify the thermodynamic limitations. The removal of H_2_O gives **7** with the carboxyl orientation similar to that in **4** formed in path **A**. The main difference between **4** and **7** lies in the distribution of H atoms in the system, and the energy difference between them effectively determines the nature of the confined extra‐framework species. Whereas **4** contains a formally +2 charged Ir_4_(H)_2_COOH cluster and a Brønsted acid site remaining at its original position, the “water‐assisted carboxyl” path **C** gives an Ir_4_(H)_3_COOH cluster in **7** with a formal +3 charge that is compensated by two vicinal lattice anions and one distant [AlO_4_]^−^ site. Such indirect charge‐compensation mechanism has been thoroughly discussed elsewhere.19a, 32

The interconversion between **4** and **7** can proceed through the reverse hydrogen spillover process, in which the adsorbed CO_2_ molecule acts as the hydrogen mediator. In the absence of CO_2_, the hydrogen spillover from the zeolite Brønsted acid sites to Ir_4_ was studied theoretically by the group of Rösch22d and it was found to be strongly exothermic by 168 kJ mol^−1^. The lower exothermicity (Δ*E*=54 kJ mol^−1^) of the reaction in our case is caused by the lower reactivity of the hydrogenated cluster bearing additional −COO and H ligands. Furthermore, the fully periodic nature of our model allowed us to consider the H transfer from a distant Brønsted acid site to the reactive Ir_4_ cluster resulting in a substantial charge alternation that contributes to the effective destabilisation of the system. The difference in the nature and formal charge of the extra‐framework iridium species in **4** and **7** results in the slight difference in the interatomic distances. The binding to a more cationic species in **7** results in a slightly longer Ir–C distance of 2.02 Å than the value of 1.98 Å in **4**. For comparison, a related molecular carboxylate Ir–PNP pincer complex^14^ has an Ir−C bond of 1.99 Å and the respective value in bulk iridium carbide systems is 2.03 Å.^33^

The hydrogenation of CO_2_ can also proceed through a mechanism involving formate species as the key intermediate commonly discussed in homogeneous systems.^3^, ^10^, ^11^, ^12^, ^13^ In the respective path **C** (Figure 2), an Ir_4_‐bound formate **8** (Ir_4_−OOCH) is formed upon the hydride attack on the carbon atom in the adsorbed CO_2_ instead of the carboxyl moiety in path **A**. This reaction (**2**→**8**) faces a moderate barrier of only 79 kJ mol^−1^, which is nearly twice as low as the one computed for the water‐free carboxyl path **A**. However, it is unlikely that the hydride attack can be facilitated by protic solvents. This is in contrast to the alternative proton‐transfer step in the carboxyl routes. This, together with the comparable energetics computed for the respective formate‐ (**C**) and water‐assisted carboxyl (**B**) paths, allow us to propose that depending on the composition of the reaction medium, both paths can take place affecting thus the selectivity of the CO_2_ reduction process. The Ir_4_–OOCH formate complex **8** can be further stabilised by 49 kJ mol^−1^ by a facile (*E* ^≠^=12 kJ mol^−1^) rotation of the formate anion resulting in species **9**.

**Figure 3:**
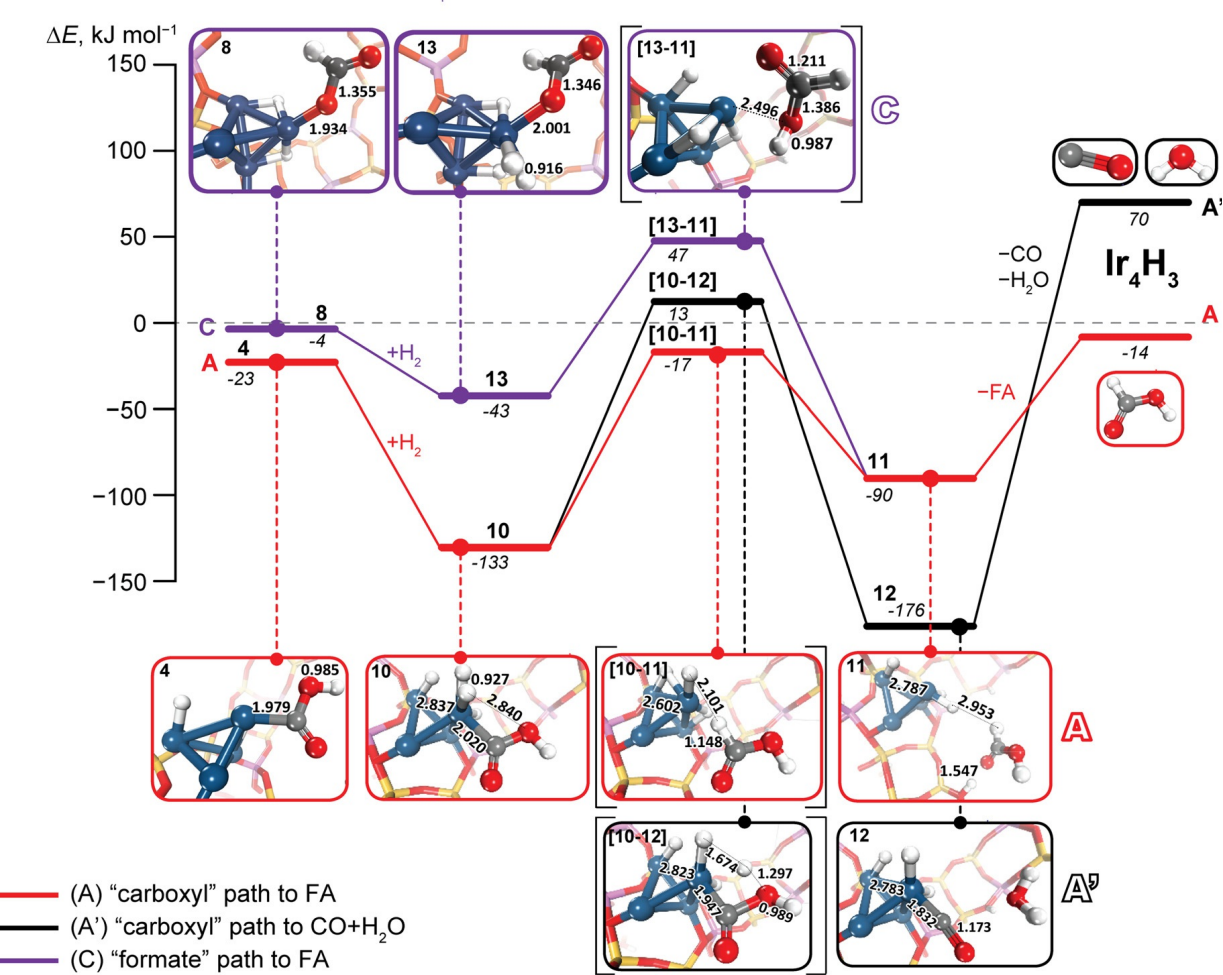
The energy diagram for the final steps of water‐free CO_2_ hydrogenation cycles on the Ir_4_ cluster. The relative energies for each state (in italic) are given in kJ mol^−1^ with respect to the sum of the energies of free reactants. The hydrogenation route of the “carboxyl path” to FA (**A**) is shown in red, and the respective reduction channel to carbon monoxide and water (**A′**) is shown in black. The “formate” path **C** towards formic acid is shown in purple. Transition‐state structures are denoted by the square brackets. The Ir cluster is represented by blue spheres and H is shown as white spheres. C, O, Si and Al are shown in grey, red, yellow and purple, respectively.

Further transformations along these paths can yield either the formic acid or CO as the final products. For the formate path **C**, our calculations reveal a single path to FA, which will be discussed below. For the carboxyl paths, the selectivity of CO_2_ conversion is determined at the next reaction stage. The associated computed reaction energy diagrams and local optimised geometries of intermediates and transition states involved are summarised in Figure 3.

Both paths to FA or CO and H_2_O require addition of an H_2_ molecule to the [Ir_4_(H)_2_–COOH]^2+^ complex in **4**. Alternative hydrogenation paths involving H transfer from the zeolite lattice or other Ir‐bound hydrides are highly unfavourable. H_2_ adsorption to the top atom of the cluster is exothermic (*E*_ads_=110 kJ mol^−1^) and yields a strongly activated *σ*‐H_2_ complex^34^ **10** with a substantially elongated H−H distance (0.93 Å vs. 0.75 Å in gas phase H_2_). Subsequent H transfer to the carboxyl ligand determines whether the formic acid (**10**→**11**, path **A**, Figure 3) or carbon monoxide and water (**10**→**12**, path **A′**, Figure 3) are formed. Hydrogenation at the O atom in COOH will lead to water formation and subsequent release of CO (**A′**: **10**→**12**→CO+H_2_O+**Ir_4_H_3_**), whereas the H transfer to the C atom in COOH directly yields FA product (**A**: **5**→**6**→FA+**Ir_4_H_3_**, Figure 3). The barrier to FA formation (*E* ^≠^=116 kJ mol^−1^) is lower than that for the decarbonylation path **A’** (*E* ^≠^=146 kJ mol^−1^) suggesting that FA is the kinetically preferred product in the current system.

A similar conclusion can be drawn from the analysis of the reaction channel via the formate intermediate **8** (path **C** in Figure 3). This catalytic path is continued by H_2_ coordination to **8** that is much less exothermic (**8**+H_2_→**13**, Δ*E*=−39 kJ mol^−1^) than the respective step in the carboxyl paths (**4**+H_2_→**10**, Δ*E*=−110 kJ mol^−1^). Nevertheless, the resulting *σ*‐H_2_ complex **13** is structurally very similar to **10**. The H−H distances in adsorbed H_2_ moieties in these structure differ by only 0.01 Å. Hydrogenolysis of the Ir−O bond in Ir−OOCH at the next step yields an FA adduct **11** common for both the carboxyl **A** and formate **C** paths. The hydrogenolysis step is exothermic by 47 kJ mol^−1^ and has a barrier of 90 kJ mol^−1^. The latter is very close to the overall barrier for the initial CO_2_ activation over this path (**1**→**8**, *E*_app_=91 kJ mol^−1^) suggesting that depending on the conditions, either of the elementary processes may become rate‐determining.15b

**Figure 4:**
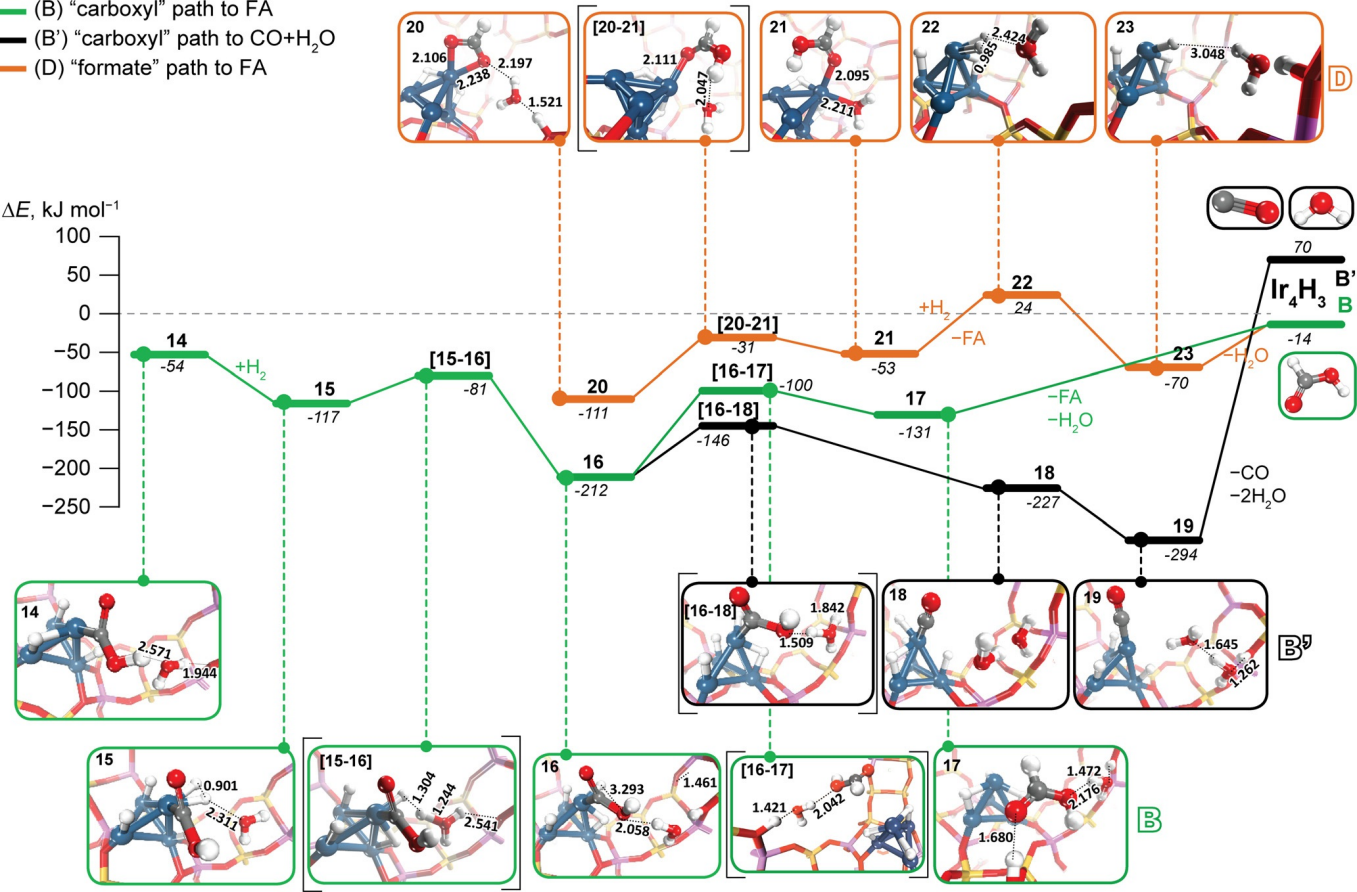
Water‐assisted paths for the hydrogenation of Ir‐bound carboxyl (**B** and **B′**) and formate (**D**) species. The relative energies for each state (in italic) are given in kJ mol^−1^ with respect to the sum of the energies of free reactants. The hydrogenation route of the “carboxyl path” to FA (**B**) is shown in green, the respective reduction channel to carbon monoxide and water (**B′**) is shown in black. The water‐assisted “formate” path **D** to FA is given in orange. Transition‐state structures are denoted by the square brackets. The Ir cluster is represented by blue spheres and H is shown as white spheres. C, O, Si and Al are shown in grey, red, yellow and purple, respectively.

In view of the strong promoting effect of co‐adsorbed water on the initial CO_2_ activation by Ir_4_H_3_/FAU, we also considered a water‐assisted path for the transformations of the resulting COOH adducts. The corresponding reaction energy diagram and optimised structures of the intermediates and transition states are shown in Figure 4. The starting point for the water‐mediated paths is structure **14** that is a [Ir_4_(H)_3_COOH]^3+^ cation with a co‐adsorbed water molecule. H_2_ adsorption to **14** (**14**→**15**) resembles the analogous process in the water‐free system (**4**→**10**). The calculated adsorption energy is 63 kJ mol^−1^. H−H bond length in the *σ*‐H_2_ complex **15** is 0.90 Å with one of the H atoms forming a hydrogen bond with the co‐adsorbed H_2_O. The heterolytic dissociation of H_2_ assisted by the co‐adsorbed H_2_O molecule regenerates the zeolite BAS, which was consumed at the initial step of CO_2_ activation (**15**→**16**). This reaction is strongly exothermic (Δ*E*=−95 kJ mol^−1^) and shows a very low activation barrier (*E* ^≠^=36 kJ mol^−1^).

The presence of H_2_O alters the selectivity trends discussed above for catalysis via **4**. Contrary to the water‐free paths (Figure 3), the formation of FA (path **B**, green line in Figure 4) is now kinetically less favourable than the decarbonylation route **A**. The activation barrier for the H transfer to the carbon atom of the adsorbed COOH resulting in FA (**16**→**17**) is 46 kJ mol^−1^ higher than the competing protonation step in path **B′** to form CO (**16**→**18**, Figure 4). However, owing to a very strong binding of CO to the Ir_4_ cluster, the regeneration of the initial active species is expected to be unlikely. One can expect that the strongly bound CO may undergo secondary hydrogenation transformations under the catalytic conditions, including the methanation reaction that is, however, beyond the scope of the current manuscript. On the contrary, the products of the direct hydrogenation path to FA do not form strong bonds with the iridium cluster and can potentially be desorbed to close the catalytic cycle. Desorption of both FA and H_2_O from **17** to regenerate Ir_4_H_3_ cluster is endothermic by only 117 kJ mol^−1^.

The formate path can also benefit from the water assistance in the transfer of the proton from the BAS. H_2_O adsorption to Ir_4_–OOCH complex **9** gives species 20 (Δ*E*=−58 kJ mol^−1^) that is the starting point for the water‐assisted reaction path (orange path **D** in Figure 4). The subsequent proton transfer from zeolite BAS to Ir‐bound formate (**20**→**21**) yields adsorbed FA. This step is endothermic by 58 kJ mol^−1^ and shows a moderate barrier of 80 kJ mol^−1^. Next, the FA product is released by an endothermic ligand exchange reaction with molecular H_2_ (**21**+H_2_→**22**+FA, Δ*E*=77 kJ mol^−1^). With the assistance of co‐adsorbed H_2_O, H_2_ undergoes a barrierless heterolytic dissociation to regenerate Ir_4_H_3_ site and BAS (**22**→**23**). The energetic cost of the removal of H_2_O from **23** (56 kJ mol^−1^) is almost identical to the value of H_2_O adsorption energy to **9**, which evidences the catalytic role of co‐adsorbed water in this reaction path (Figure 4).

These computational results suggest the possibility of controlling the reaction thermodynamically and kinetically. The formation of CO occurs with a barrier of only 66 kJ mol^−1^, but leads to a very stable system from which the products desorption (CO and 2 H_2_O molecules) faces a prohibitive energy penalty of 366 kJ mol^−1^. The high endothermicity of this step is the result of a strong binding of CO with metals. For example, the experimentally determined CO adsorption energy to Ir (1 1 1) surface is 175 kJ mol^−1^.^35^

In view of such a strong adsorption of CO, its further hydrogenation to obtain methanol, methane or even to promote the C−C bond formation upon the coordination to the active Ir_4_ site additional CO_2_ molecule cannot be excluded. Those anticipated products would bind weaker with the Ir cluster and as such, their release may become facilitated. The detailed computational analysis of such paths requires a separate dedicated investigation that is beyond the scope of this study.

Our findings suggest that by varying the concentration of water in the reaction medium one can potentially selectively promote the FA synthesis by favouring the formate route or by lowering the kinetic barriers needed for the initial formation of the −COOH species in the carboxyl path. However, at high H_2_O concentrations, the path towards CO formation would become strongly favoured resulting in a rapid deactivation of the catalyst or drastic selectivity change towards the CO hydrogenation products. Analysis of the theoretical results indicates the possibility of choosing the preferred reaction path through variation of the experimental conditions and by performing the hydrogenation with simultaneous water removal or at very low water content.

## Conclusions

CO_2_ hydrogenation over zeolite Y‐supported Ir_4_H_3_ clusters was studied by periodic DFT calculations utilising a realistic faujasite model to simulate a realistic zeolite environment adequately. The computational results allowed us to identify two possible reaction paths for the CO_2_ reduction, namely the direct hydrogenation towards formic acid and reduction to CO and H_2_O. The reactivity of the system and the reaction selectivity greatly depends on the presence of water molecules in the system, which act as proton mediators promoting the proton‐transfer reactions along the catalytic cycles. The promotion effect of water on hydride transfers to form C−H bonds is low.

The competing paths are interrelated in an yin–yang manner because water molecules produced along the decarbonylation path can in situ modify the preferred reaction route. Under the water‐free conditions, the initial CO_2_ hydrogenation to Ir‐bound COOH species shows a barrier of 143 kJ mol^−1^ that is substantially higher than the alternative path to form the Ir–OOCH formate adducts, which are converted further into FA exclusively. However, the initial formation of Ir–COOH carboxyl moiety can be greatly facilitated in the presence of water that mediates a proton transfer from the zeolite lattice to the adsorbed CO_2_. Water reduces the barrier for the COOH formation by approximately 40 kJ mol^−1^ and makes the respective reaction channel competitive with the formate path. The co‐adsorbed water promotes the selective production of CO by an H_2_O‐assisted decarbonylation of COOH species through reduction of the corresponding activation barrier by a similar value of 46 kJ mol^−1^ with respect to the competing hydrogenation path to FA.

We conclude that it is virtually impossible to obtain the formic acid in an aqueous environment by using the Ir/FAU catalyst, and an effective in situ water‐removal has to be ensured if the selectivity towards FA is desired. By performing the reaction under H_2_O‐free conditions, the decarbonylation path can be suppressed. This is necessary in view of the very strong binding of CO product to the active Ir_4_ cluster (*E*_ads_=246 kJ mol^−1^). On the other hand, the desorption of formic acid is much easier (*E*_ads_=94 kJ mol^−1^). We propose that by optimizing process conditions, its selective formation can potentially be achieved by the liquid‐phase hydrogenation of CO_2_ over an Ir/FAU catalyst.
